# HIV Protein TAT Dysregulates Multiple Pathways in Human iPSCs-Derived Microglia

**DOI:** 10.3390/life15071082

**Published:** 2025-07-09

**Authors:** Liam Liyang Guo, Robert Jiang, Yan Cheng, Brooke Russell, Sanders Y. Yan, Ming-Lei Guo

**Affiliations:** 1 Keck Medical School, University of Southern California, Los Angeles, CA 90007, USA; liyangguo231@gmail.com; 2 Department of Biological and Translational Department, Eastern Virginia Medical School/Virginia Health Sciences, Old Dominion University, Norfolk, VI 23507, USA; jiang.drake@gmail.com (R.J.); chengy@evms.edu (Y.C.); b1russel@evms.edu (B.R.); sandery@evms.edu (S.Y.Y.); 3 Center for Integrative Neuroscience and Inflammatory Diseases, Eastern Virginia Medical School/Virginia Health Sciences, Old Dominion University, Norfolk, VI 23507, USA

**Keywords:** NeuroHIV, lipid droplet accumulation microglia, transactivator of transcription, human inducible pluripotent stem cells, microRNAs

## Abstract

In the era of combined antiretroviral therapy, around 50% of chronic HIV (+) individuals show varying degrees of memory and cognitive deficiency (NeuroHIV), a phenomenon of accelerated brain aging. HIV protein transactivator of transcription (TAT) has been well-accepted as a risk factor contributing to NeuroHIV through dysregulating microglia (Mg) functions. Previous studies have demonstrated that HIV-TAT can affect lipid metabolism, immune responses, autophagy, and senescence in rodent Mg. However, due to the significant species differences between rodent and human Mg (hMg), it is essential to take caution when interpreting the results obtained from rodent models into human conditions. For the unanswered questions, we generated hMg from human inducible pluripotent stem cells (iPSCs) and exposed them to HIV-TAT. The results obtained from Flow analysis and immunostaining experiments reveal that TAT can induce LD accumulation and increase perilipin-2 (Plin2) levels in hMg. Meanwhile, HIV-TAT can upregulate autophagosome formation and p53 levels. Through human immune array assay, we showed that TAT can increase the expression of multiple pro-inflammatory mediators, cytokines, and chemokines in hMg. Extensive bioinformatic analysis shows that HIV-TAT can affect multiple neuroimmune signaling pathways and indicates that microRNAs (miRNAs) are coherently involved in such dysregulation. Overall, our findings provide direct evidence showing that HIV-TAT can affect lipid metabolism, autophagy, senescence signaling, and multiple neuroimmune-related pathways in hMg and indicate the roles of novel miRNAs on NeuroHIV pathogenesis, which deserves further investigations.

## 1. Introduction

In the era of combined antiretroviral therapy (cART), people living with HIV can live longer than in the pre-cART time, and their life-expectancy is comparable to HIV (−) population [1]. About 50% of people living with HIV (PLWH) in the USA are above 50 years old [2]. However, the life quality of PLWH is deeply compromised due to the accelerated aging process with higher penetrance of aging-related diseases, including neurocognitive and memory deficiencies, cardiovascular diseases, diabetes, and fragile [3,4,5,6,7]. Epidemiological investigations show that up to 50% of chronic HIV (+) individuals can be diagnosed with varying degrees of memory and cognitive impairments (NeuroHIV) [8]. The brains with NeuroHIV are characterized by abnormal microglia (Mg) activation (elevated neuroinflammation) and neuronal injuries [9]; Mg dysregulation has been well-accepted as a driving force promoting NeuroHIV pathogenesis [8,9].

Microglia, the brain’s resident macrophages, determine neuroinflammation levels in response to internal and external stimuli. Mg can be classically categorized into M0 (quiescence), M1 (pro-inflammatory), and M2 (anti-inflammatory) status in the brain [10,11]. However, the concept of Mg has been significantly advanced in the past decade due to the breakthrough in the single-cell RNA (scRNA) sequencing technique [12,13]. Mg are heterogeneous in the brain under both physiological and pathological states [14,15]. Mg can be classified into several subtypes based on their expression profile. A novel subset of Mg called disease-associated microglia has been identified in the brains of mouse models with Alzheimer’s disease or with Parkinson’s disease [16,17]. Proliferation-associated microglia, neurodegeneration-associated microglia, lipid-droplet-accumulation microglia (LDAM), etc., have also been identified in various disease models [18,19,20]. Among these subtypes, LDAM have drawn particular attention due to their critical roles in promoting the brain aging process and pathogenesis of neurodegenerative disease [21,22,23]. At the cellular level, LDAM are characterized by phagocytosis deficiency, excessive secretion of proinflammatory cytokines, high reactive oxygen species levels, and reduction in cholesterol efflux [23,24,25]. LDAM have been characterized as similar to senescent Mg [26,27]; still, the exact correlation between these two types of Mg needs further investigation [23,28].

HIV proteins, including transactivator of transcription (TAT), can be persistently expressed in the brains of HIV (+) individuals, although antiretrovirals inhibit HIV replication in vivo [29]. HIV-TAT has been accepted as a major risk factor contributing to Mg activation and NeuroHIV pathogenesis. Previous studies have shown that TAT can affect multiple pathways in rodent Mg, including autophagy, senescence, NLRP3 inflammasome, etc. [30,31,32]. Our recent investigations show that HIV-TAT can increase LDAM in the brains of HIV-transgenic rats and mice [33,34]. However, one should be cautious about extrapolating the findings obtained from rodent Mg into hMg due to huge species differences in every aspect of biology [35,36,37]. Mg derived from rodent and human cells show distinctive behaviors in their proliferation and adhesive ability to the plate in vitro culture [38,39]. For example, TGFβ1 could block interferon-γ-mediated HLA expression in rodent Mg, while these effects could not be observed in human Mg [40,41]. Toll-like receptor 4 (TLR4) is at relatively lower levels in human Mg compared to rodent Mg, indicating that this inflammatory pathway relevant to hMg activation might not be as relevant as to rodent Mg [42,43]. Also, previous studies showed that nitric oxide can be readily produced in rodent Mg in response to inflammatory stimuli, but not in human Mg [44,45]. So there exists a big difference in the signal transductions in response to stimuli between human and mouse Mg. Therefore, it is obligatory to validate the findings obtained from rodent Mg in human counterparts.

In this investigation, we want to verify whether HIV-TAT can induce similar biological effects in hMg as in rodent Mg. Currently, there are several approaches to obtain human Mg, such as human primary Mg culture, embryonic stem cell- or iPSC-derived Mg, with advantages and disadvantages. Among these three approaches, iPSC-derived Mg can provide a large number of cells carrying an adult microglial phenotype [46]. The application of this technique has been generating novel insights into the pathogenesis of neurodegenerative disorders, including Alzheimer’s disease [47,48,49]. In this study, we employ this advanced technique to explore whether HIV-TAT can affect LDs formation, the autophagy process, as well as senescence in hMg. Our findings provide direct evidence demonstrating that HIV-TAT can induce similar changes on multiple cellular pathways in hMg as in rodent Mg, and these pathways are indeed involved in NeuroHIV pathogenesis.

## 2. Materials and Methods

Reagents: We purchased HIV-TAT recombinant protein from ImmunoDox (Product no. 1032, Woburn, MA, USA); the protein was dissolved in PBS, aliquoted, and stored at −80 °C. We purchased BODIPY™ 493/503 (D3922) from Thermo Fisher Scientific (Waltham, MA, USA) and dissolved it in DMSO at a stock concentration of 2 mM. BODIPY was used as a working solution at 2 µM. Human iPSC lines were purchased from SAMPLED company (Piscataway, NJ, USA). The maintenance and propagation of human iPSCs were performed based on the protocols recommended by STEMCELL Technologies, Vancouver, BC, Canada.

Generation of hMg: We utilized a two-step protocol recommended by the company to generate hMg (STEMCELL Technologies). Human iPSCs were first seeded into 6-well plates with TeSR^TM^ medium (Cat #100-0276) containing ROCK inhibitors (Cat #1254, Tocris, Bristol, UK) overnight. The next day, TeSR^TM^ was replaced by Medium A (STEMdiff^TM^ Hematopoietic Basal Medium with supplement A, Cat #100-0171) for three days, with the half medium change on the second day. On the fourth day, Medium A was replaced by Medium B (STEMdiff^TM^ Hematopoietic Basal Medium with supplement B, Cat #100-0173) for 12 days, with half Medium B change every other day to differentiate into hematopoietic progenitors (HPCs). Following this, HPCs were digested and seeded into another 6-well plate with serum-free microglia differentiation medium (Cat #100-0019) containing human recombinant MCSF, IL34, TGFβ1, and insulin for 24 days. Half differentiation medium was added every other day. On the 25th day, the differentiation medium was replaced by STEMdiff™ Microglia Maturation medium (Cat #100-200), with adding human recombinant CD200 and CX3CL1, and cultured for another 7 days. The hMg were then stained with PE Mouse Anti-Human CD11b (BD Pharmingen™, Cat #555388) and FITC Mouse Anti-Human CD45 (BD Pharmingen™, Cat #555482) antibodies to confirm their maturation status.

BODIPY staining: We seeded mature hMg into the chamber slides overnight with 10% FBS DMEM. At the indicated time after HIV-TAT/PBS treatments, hMg were washed with PBS and incubated with BODIPY solution (2 µM) for 15 min at 37 °C. The cells were washed three times with PBS wash, followed by 4% PFA for 30 min at room temperature. The hMg were washed with 1XPBS three times to remove the PFA. Then, the cells were covered by a coverslip with ProLong Gold antifade reagent with 4,6-diamidino-2-phenylindole (Thermo Fisher Scientific, Waltham, MA, USA, P36935). The slides were kept at 4 °C overnight; fluorescent images were acquired on a Zeiss AXIO inverted LED fluorescent observer.

Flow analysis: Mature hMg were seeded into 24-well plates exposed to varying doses of HIV-TAT (25–100 ng/mL) or PBS for 24 h. The rationale for choosing the above-mentioned TAT concentration is based on previous reports and the fact that the concentration of TAT protein in the CSF is about 16 ng/mL, while that in the serum of HIV+ patients ranges from ~0.1 to 40 ng/mL, with actual concentration at tissue sites being even higher [50,51,52]. Then, the cells were washed with PBS and incubated with BIDIPY solution (2 µM) in the dark for 15 min at 37 °C. The cells were rinsed with PBS and trypsinized to generate a single-cell suspension in 5 mL PBS. The cells were centrifuged at 250× *g* for 5 min at 4 °C twice and resuspended in 300 µL 1× flow cytometry buffer. We performed analysis with the BD Accuri™ C6 Plus Flow Cytometer System (BD company, Franklin Lakes, NJ, USA) and around 10,000 cells in each condition were included for analysis by the FlowJo software (V10).

Immunostaining: Mature hMg were seeded into chamber slides overnight. The next day, hMg were exposed with HIV-TAT (50 ng/mL) or PBS for 24 h followed with incubation with the primary anti-Plin2 antibody (1:300, Proteintech, Rosemont, IL, USA, Cat #15349), anti-LC3II antibody (1:500, Novus company, Chesterfield, MO, USA, NBP100-2220), or p53 antibody (1: 250, Proteintech, Cat #60283-1-lg) overnight at 4 °C. Secondary AlexaFluor 488 goat anti-rabbit IgG (A-11008) or AlexaFluor 594 goat anti-mouse (A-11032) (Thermo Fisher Scientific Waltham, MA, USA) was added for 2 h at room temperature. Then, hMg went through a PBS wash, mounted on slides with ProLong Gold antifade reagent. We acquired fluorescent images on a Zeiss Observer. Zenpro software (Zen 3.11, Carl Zeiss, Thornwood, NY, USA) and ImageJ (1.54K) were used to process and analyze the intensity of Plin2, p53, and LC3 signals and lipid droplets. For the fluorescence intensity quantification, we selected around 100 cells from three slides of each group (PBS- or TAT-treated). Images were obtained under the same exposure conditions.

RNA extraction, reverse transcription: Briefly, 1 × 10^6^ hMg were added in 1 mL Trizol for lysis (Cat #15596026, Invitrogen, Waltham, MA, USA). After 30 min, the lysates were sonicated for around 3–5 s and incubated for 10 min on ice. Then, the lysates were aspirated into a new 1.5 mL microcentrifuge tube, and 0.2 mL of chloroform was added. The samples were vigorously vortexed and centrifuged at 10,000× *g* for 15 min at 4 °C. We transferred the upper aqueous phase to a new tube and added 500 µL of isopropyl alcohol. The tube was incubated for 10 min and centrifuged again for RNA precipitation. The total RNA was dissolved in DEPC-treated H_2_O and quantified. We performed reverse transcription reactions using Verso cDNA kit (Cat #AB1453A, Invitrogen). We set the reaction system with 4 µL 5× cDNA synthesis buffer, 2 µL dNTP mix, 1 µL RNA primer, 1 µL RT enhancer, 1 µL Verso enzyme mix, total RNA template 1200 ng, and a variable volume of water filled up to 20 µL. Reaction conditions were set at 42 °C for 30 min.

Inflammation array and bioinformatic analysis: An Applied Biosystems TaqMan™ Array human inflammation 96-well Plate was purchased from Thermo Fisher Scientific (Cat #4414074). This plate contains 92 human inflammation-associated genes and 4 assays to identify candidate endogenous control genes. The genes fall into 4 classes: (1) channels (L-type calcium and ligand gated); (2) enzymes and inhibitors (lipases, kinases, nitric oxide synthase, phosphodiesterase, prostaglandin metabolism, and proteases); (3) factors (tumor necrosis factors, nuclear factor Kappa-Β, interleukin, annexins, and kininogens); (4) receptors (GPCR, IL receptor family, adhesion molecules, TNF receptors and nuclear receptors). Total RNA extracted from control and HIV-TAT-treated hMg (1200 ng) were used for 96-well array analysis. After reverse transcription, the cDNA was diluted and aliquoted into each reaction mixture (20 µL) for qPCR in the QS3 qPCR machine (Invitrogen) for program running. The calculations for the up- or down-fold of each gene were analyzed based on internal controls. The differentially expressed genes (DEGs) that show folds above 1.15 or below 0.8 were included for further bioinformatic analysis. The bioinformatic tools include integrated pathways analysis (IPA), Gene Ontology (GO), Search Tool for the Retrieval of Interacting Genes (STRING), and Molecular Complex Detection (MCODE). Cytoscape (3.10.3) was used for DEGs-miRNAs interaction and to identify the transcriptional network in the analysis. We validated the array results by performing individual qRT-PCR, and the primers and probes were purchased from Thermo Fisher Scientific. GAPDH was selected as an internal control.

Statistical analysis: The statistical data are expressed as means ± the standard error of the mean (SEM). Data were analyzed using two-tailed Student’s *t*-test, one-way analyses of variance (ANOVA) procedures followed, when appropriate, by Tukey–Kramer multiple comparisons tests using GraphPad Prism 8 (La Jolla, CA, USA). We considered probability levels of <0.05 as statistically significant. For analysis on immunofluorescence signal intensity, we included around 100 hMg from PBS- or HIV-TAT-treated groups.

## 3. Results and Discussion

**Figure 1:**
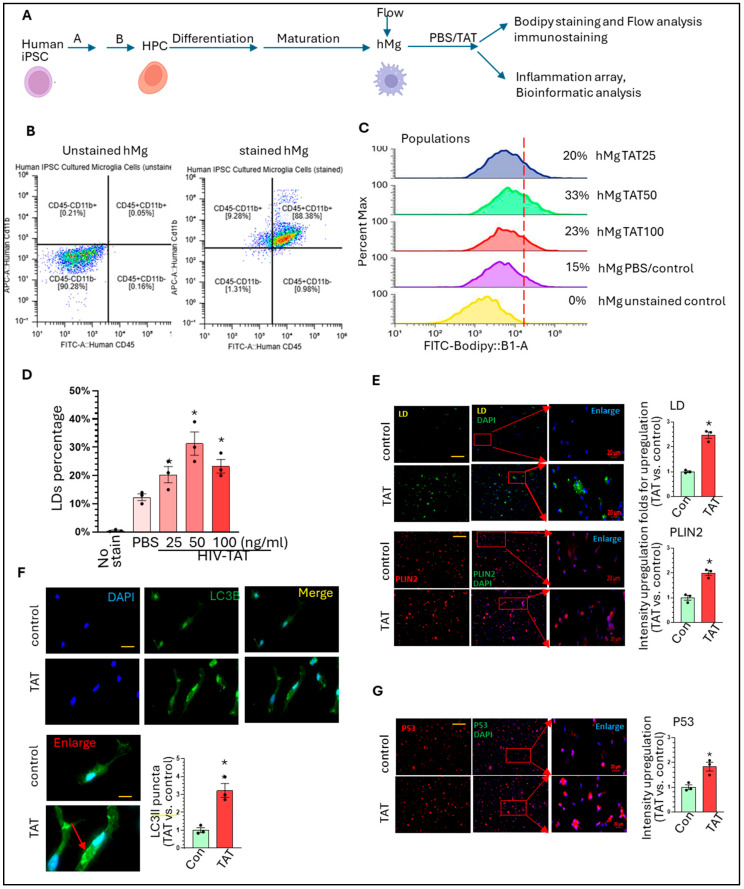
HIV-TAT dysregulates lipid metabolism, autophagy, and senescent pathways in hMg: (**A**) Brief schematics for hMg generation from human iPSCs; (**B**) The results show that >90% of hMg are CD11b^+^CD45^+^; (**C**) HIV-TAT increases BODIPY positive hMg at varying doses; (**D**) HIV-TAT increases LDs accumulation in hMg (F (4, 10) = 21.13, one-way ANOVA, * *p* < 0.05, TAT treatment groups vs. PBS group). (**E**) HIV-TAT increases the numbers and intensity of LDs and Plin2 levels in hMg (LD: green; DAPI, blue, Plin2: red; LD and Plin2: unpaired two-tailed Student-*t* test, TAT vs. control, * *p* < 0.05). (**F**) HIV-TAT enhances the number of LC3B dot-like puncta (autophagosome marker) in hMg (DAPI: blue; LC3B: green; unpaired two-tailed Student-*t* test, TAT vs. control * *p* < 0.05). (**G**) HIV-TAT increases p53 levels in hMg (P53: red; DAPI: blue; unpaired two-tailed Student’s *t*-test, TAT vs. control * *p* < 0.05). Three batches of hMg were subjected to these experiments or statistical analysis. Scale bar 20 µm or 10 µm. Around 100 hMg from PBS- or HIV-TAT-treated groups were included for calculating the intensity of immunofluorescence signal or the number of LC3II-formatted puncta.

HIV-TAT increases LDs accumulation, autophagosome formation, and P53 levels in hMg: We established a platform to generate hMg from human iPSCs based on recommended protocols (schematic in Figure 1A). On the day of maturation, hMg were collected for CD11b and CD45 double immunostaining, followed by Flow analysis to confirm their identity (Figure 1B, >90% are CD11b^+^CD45^+^). We seeded hMg into 24-well plates and treated them with PBS (control) or varying doses of HIV-TAT (25–100 ng/mL) for 24 h. Of note, HIV-TAT has the capacity to enter the cells from the outside medium [53,54]. We stained hMg with Bodipy and determined the percentages of LDs positive hMg in each condition by Flow analysis. The unstained hMg were taken as control for setting up gating parameters and showed 0% LDs positive. hMg with PBS treatment show around 15% of LDs positive (basal levels). Interestingly, hMg with TAT treatment show increased percentage of LDs positive (25 ng/mL, ~20%; 50 ng/mL, ~33%; and 100 ng/mL, ~23%) which show that the maximum effects of TAT is on 50 ng/mL (Figure 1C,D, * *p* < 0.05, one-way ANOVA, experiments were repeated three times for analysis). We selected this dose for later experiments. The decreased percentages of LDs for hMg with 100 ng/mL are probably due to the toxic effects of TAT [55]. To consolidate the effects of TAT on LDs formation, we performed Bodipy or Plin2 immunostaining in hMg with HIV-TAT (50 ng/mL) or PBS treatments. The results show that TAT significantly increased LDs formation (* *p* < 0.05, 2.47 ± 0.21 folds) and Plin2 levels (* *p* < 0.05, 1.99 ± 0.17 folds) (Figure 1E, two-tailed Student’s *t*-test). Since autophagy dysregulation is implicated in LDs formation in Mg [56,57], we explored whether TAT could dysregulate the autophagy process in hMg, similar to the process observed in rodent Mg [58]. We performed LC3B immunostaining to check the status of LC3B-formed puncta (autophagosome marker) in TAT or PBS-treated hMg. Our results show that TAT could significantly upregulate LC3B dot-like puncta, implying that HIV-TAT can dysregulate the autophagy process (Figure 1F, * *p* < 0.05, two-tailed Student’s *t*-test, 3.25 ± 0.39 folds). LDAM have been suggested as senescent Mg, we then check the levels of p53 (senescent marker) in these two groups and the results showed significant upregulation on p53 levels in the TAT-treated group (Figure 1G, * *p* < 0.05, two tailed Student’s *t*-test, 3.25 ± 0.39 folds) meaning potential senescence. Overall, these findings indicate that HIV-TAT can induce LDs formation, autophagy dysregulation, and potentially promote senescence in hMg, similar to the effects observed in rodent Mg, as shown in previous publications [30,31,34,59].

**Figure 2:**
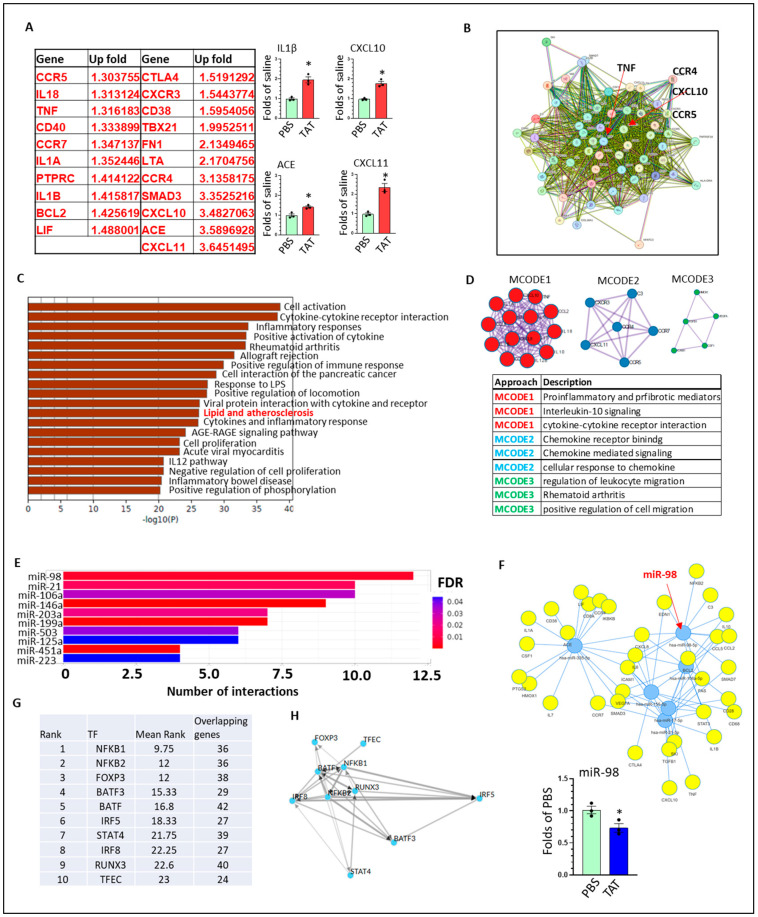
HIV-TAT upregulate inflammatory related factors in hMg: (**A**) array results show genes with up- (1.3 folds) or down-regulated (<0.8 folds) by HIV-TAT in hMg; qRT-PCR verify the regulation effects of TAT on IL1β, CXCL10, ACE, and CXCL11 in hMg (unpaired two-tailed Student’s-*t* test, TAT vs. control, * *p* < 0.05); (**B**) The interaction map of these DEGs; (**C**) IPA analysis show enriched pathways dysregulated by HIV-TAT in hMg; (**D**) MCODE show top enriched signaling upregulated by HIV-TAT; (**E**) Top10 miRNAs that are identified to be responsible for DEGs; (**F**) The miRNAs-DEGs map highlighting with miR-98. QRT-PCR results show that HIV-TAT significantly decreases miR-98 levels in hMg (unpaired two-tailed Student’s *t*-test, TAT vs. control * *p* < 0.05); (**G**) The top 10 transcriptional factors that are identified for DEGs; (**H**) the interaction map of the top 10 transcriptional factors. For qRT-PCR validation, each group contains three independent samples for statistical analysis.

HIV-TAT upregulates multiple cytokines, cytokine receptors, and pro-inflammatory mediators in hMg: To explore the effects of HIV-TAT on inflammatory-related pathways, we treated hMg with TAT (50 ng/mL, 24 h) or PBS, followed by total RNA extraction. We compared the expression profile of 92 inflammatory-related genes between the TAT- and control groups by using a human inflammation array. The GAPDH was used for quantity control. The results show that 39 genes were upregulated by at least 1.15 folds and 21 genes by at least 1.3 folds up (*p* < 0.05). On the other hand, five genes were down-regulated (<0.8 folds, *p* < 0.05) by HIV-TAT (Figure 2A). We performed individual qRT-PCRs to validate the upregulation effects of TAT on four genes, including IL1β, CXCL10, angiotensin-converting enzyme (ACE), and CXCL11 (Figure 2A). We observed that classical pro-inflammatory mediators IL18 (1.31 up), TNF (1.31 up), IL1α (1.35 up), and I L1β (1.41 up) are among the list, indicating that hMg are activated by HIV-TAT. Since they all can serve as signal 1 for NLRP3 priming, we reasonably assume the NLRP3 pathway is primed in TAT-exposed hMg, which validates the critical roles of NLRP3 inflammasome in NeuroHIV. CXCR3, CXCL10, and CXCL11 belong to the chemokine superfamily and have been extensively investigated during HIV pathogenesis [60,61,62]. The upregulation of these three chemokines indicates that HIV-TAT can directly stimulate hMg to release chemokines, attracting monocytes from the peripheral system into the brain. Another interesting observation is that CCR5, the co-receptor for HIV infection, can be directly upregulated by TAT in hMg, which may help HIV infection. The direct upregulation effect of HIV-TAT on ACE has never been shown. ACE can convert angiotensin I into angiotensin II, which is a potent vasoconstrictor, increasing blood pressure. Chronic HIV (+) individuals show high penetrance of cardiovascular diseases and hypertension [63,64]. Our results imply that ACE is probably involved in this comorbidity, and targeting ACE might provide therapeutic effects for cardiovascular diseases in chronic HIV (+) individuals.

To acquire more information from this human array, we performed bioinformatic analysis on these DEGs using varying bioinformatic tools. These DEGs can interact with each other to institute a complex regulation map, indicating that these molecules can modulate each other and composite a network to determine the activation status of hMg (Figure 2B). For identifying pathways relevant to the DEGs, we performed IPA analysis, and the results show that cell activation, cytokine–cytokine receptor interaction, and inflammatory responses are the top three enriched pathways, which is consistent with the results obtained from rodent-derived Mg (Figure 2C). We also found the lipid and atherosclerosis pathway in the IPA list, which supports the notion that dysregulation of lipid metabolism is inherently involved in TAT-mediated Mg activation. MCODE is another tool to obtain pathways related to DEGs, and we acquired similar results (Figure 2D). To search for mechanisms responsible for these DEGs, we resorted to microRNA (miRNA) epigenetic regulation. We identified the top 10 miRNAs that can interact with these DEGs with a false discovery rate (FDR) < 0.05 (Figure 2E). Among them, miR-21, miR-146a, miR-199a, and miR-223 are well-known for their roles in HIV pathogenesis, which validates our bioinformatic results [65,66,67,68]. Interestingly, miR-98 (the No. 1 in the list) has not been shown to have any role in Mg activation and HIV pathogenesis, which could be an interesting topic for further investigation. We also presented the interaction map between DECs-miRNAs, highlighting the potential role of miR-98. We individually performed qRT-PCR and confirmed that HIV-TAT significantly decreases miR-98 levels in hMg (two-tailed Student’s *t*-test, * *p* < 0.05) (Figure 2F). This result is consistent with previous investigations, implying that miR-98 is an anti-inflammatory mediator [69] and downregulates immune responses [70]. We also explore which transcriptional factors can be involved in these DEGs’ regulation and identify the top 10 transcriptional factors. NF-кB1/2 are in the top two positions, which have 36 overlapping genes (Figure 2G). We observe that interferon regulatory factors 5 and 8 might play critical roles in these DEGs, indicating the activation of the innate antiviral immune system during HIV infection. Actually, these top 10 transcriptional factors do not act alone; on the contrary, they interact with each other and institute a mutually interacting network that coordinately decides Mg immune responses (Figure 2H).

The investigations on the effects of HIV on human iPSC-derived Mg or brain organoids are just at the embryonic stage. Previous investigations have shown that HIV infection can cause hMg activation and neuronal injuries in Mg-containing brain organoids [71,72]. Activation of Toll-like receptor 3 was identified as innate immunity against HIV infection in human iPSC-derived Mg [73]. We argue that direct usage of HIV-TAT could be of more clinical significance since (1) HIV replication can be significantly inhibited and HIV proteins such as TAT is still expressed in chronic HIV (+) individuals with cART and (2) the direct effects of HIV-TAT on hMg have never been explored before. The novelty of this investigation is that we show direct evidence supporting that HIV-TAT can exert similar effects on multiple pathways on human iPSC-derived Mg including LDs accumulation, autophagy dysregulation, and senescence in hMg as in rodent-derived Mg. Also, TAT-mediated upregulation on multiple pro-inflammatory mediators, chemokines, and cytokines has been confirmed. In summary, our results verify the similar biological effects of HIV-TAT on hMg as on rodent Mg and also provide novel insights into miRNAs in NeuroHIV pathogenesis. The potential roles of miR-98 in the regulation of Mg activation deserve further investigation.

## Data Availability

The original contributions presented in this study are included in the article. Further inquiries can be directed to the corresponding author.
